# Supplementary figures and images for: Canagliflozin Promotes Structural and Functional Changes in Proximal Tubular Cell Mitochondria of Hypertensive–Diabetic Mice
Source: Int J Mol Sci. 2025 Dec 12;26(24):11988. doi: 10.3390/ijms262411988 (PMC12732475; doi:10.3390/ijms262411988)

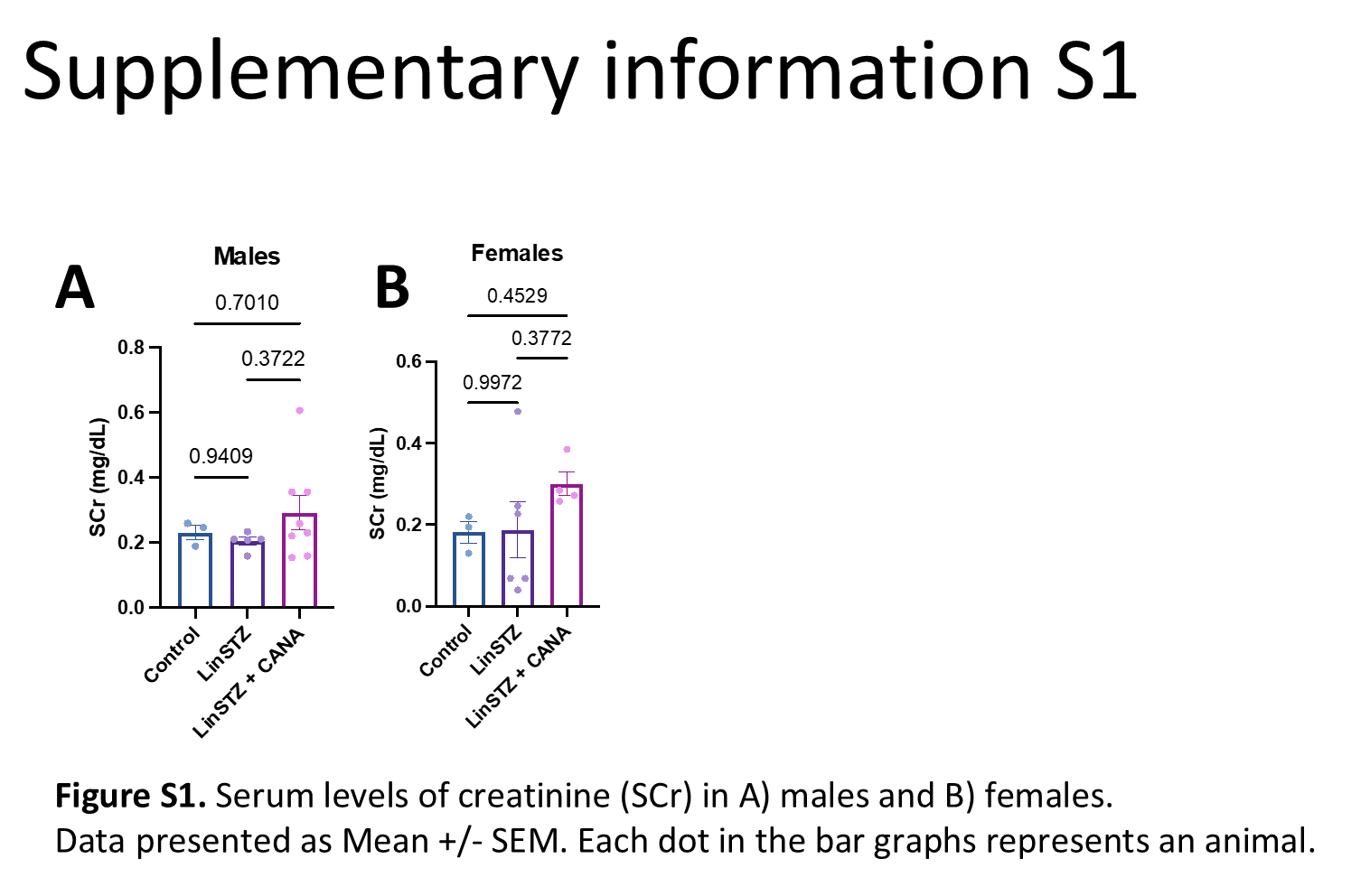

Supplement: Supplementary file 1 [file ijms-26-11988-s001.zip › Figure S1.tiff]

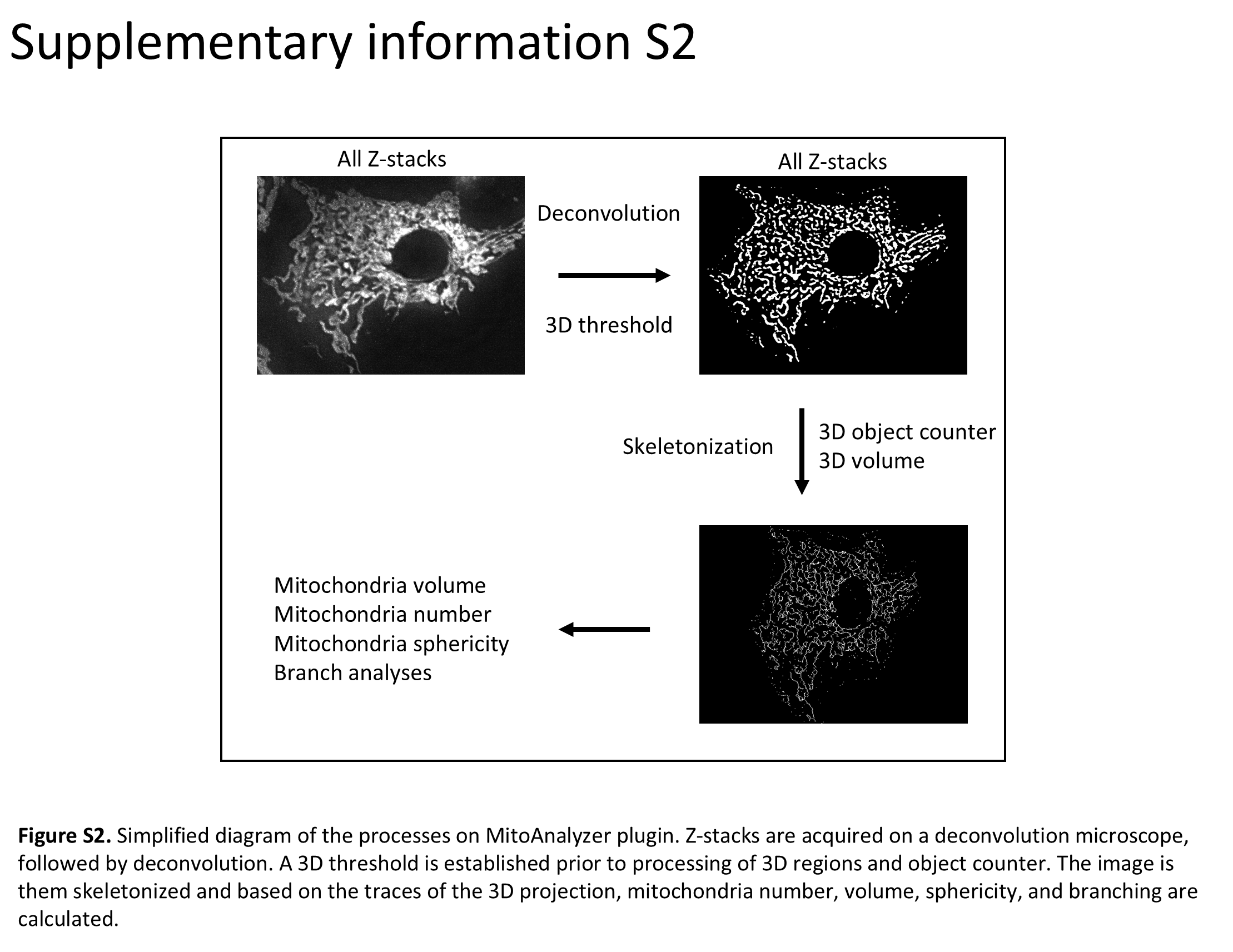

Supplement: Supplementary file 1 [file ijms-26-11988-s001.zip › Figure S2.tiff]

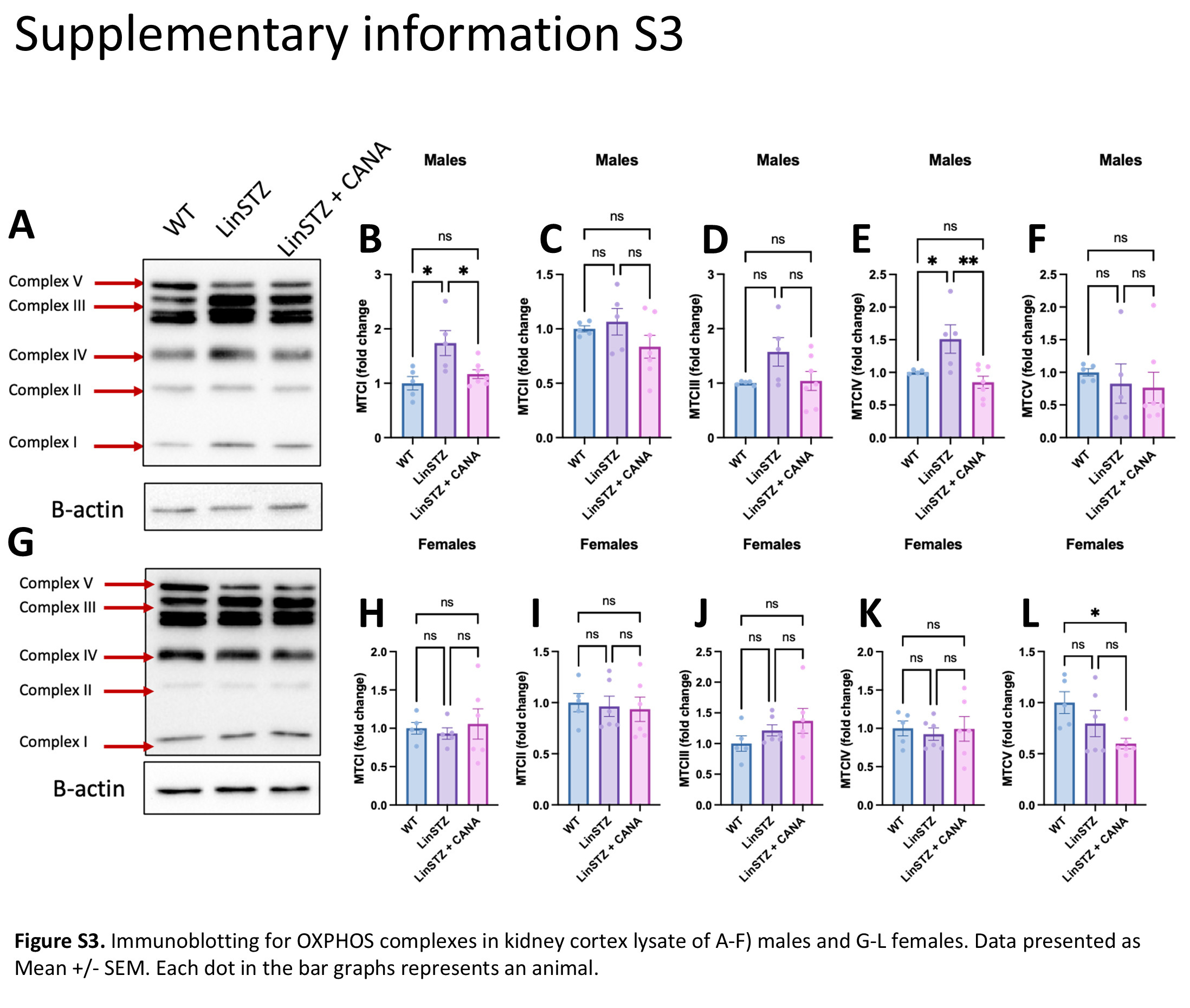

Supplement: Supplementary file 1 [file ijms-26-11988-s001.zip › Figure S3.tiff]

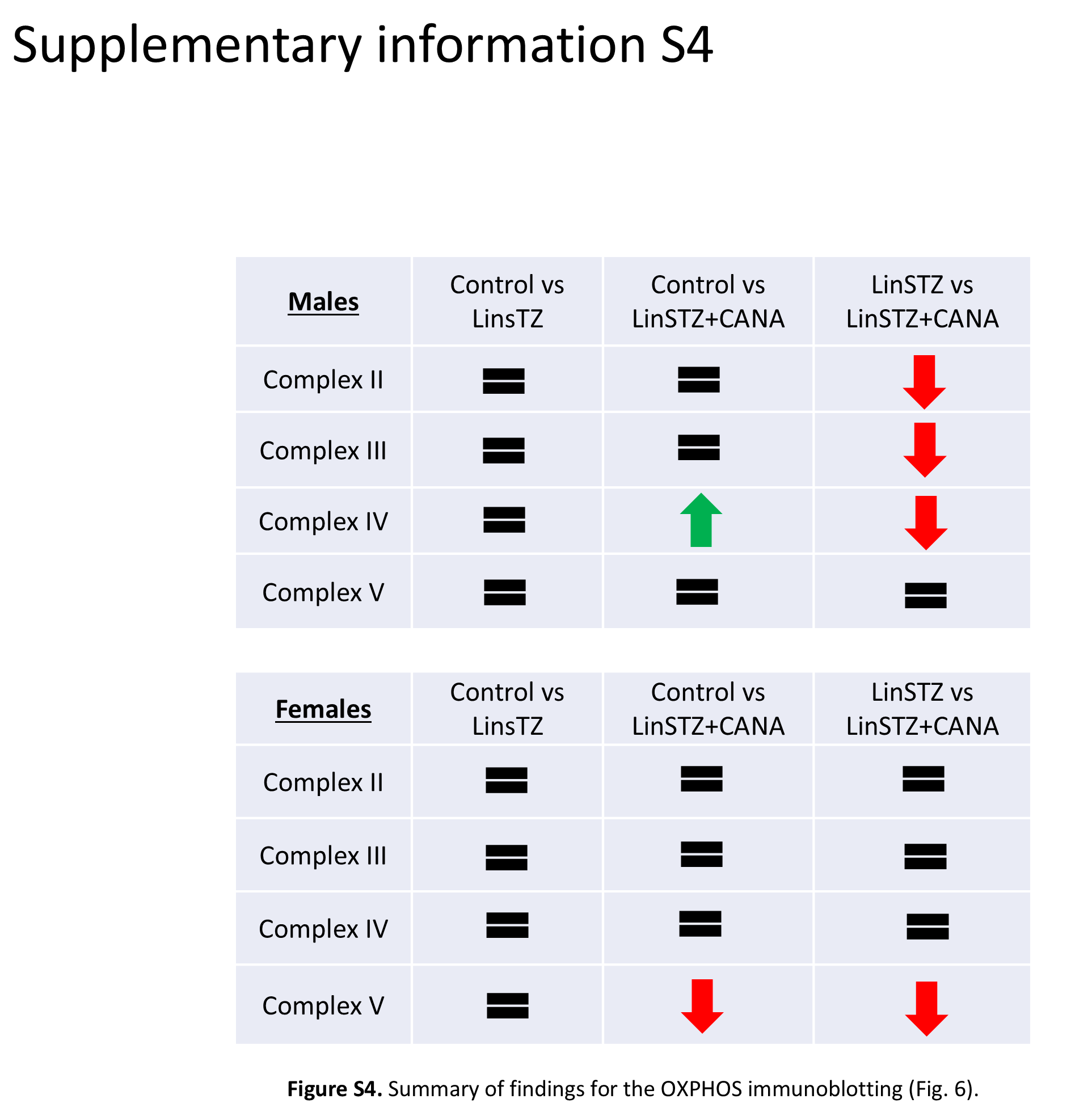

Supplement: Supplementary file 1 [file ijms-26-11988-s001.zip › FIgure S4.tiff]
